# Quantification and characterization of manufactured nanomaterials shed from face masks

**DOI:** 10.1038/s41598-025-34482-6

**Published:** 2026-02-03

**Authors:** R. Mehri, Z. Gajdosechova, T. A. Sipkens, G. J. Smallwood, A. M. Belknap, D. Vladisavljevic, J. C. Corbin

**Affiliations:** 1 Metrology Research Centre, National Research Council Canada, Ottawa, Canada; 2 New Substances Assessment and Control Bureau, Health Canada, Ottawa, Canada

**Keywords:** Particle shedding, Face mask, Manufactured nanomaterial, Titanium dioxide, Environmental sciences, Health care, Materials science, Microbiology, Nanoscience and technology

## Abstract

Face masks are an important public health measure whose use became wide-spread during the pandemic. Manufactured nanomaterials (MNMs) have been incorporated into face masks to enhance their anti-microbial and self-cleaning properties. However, the potential toxicity of certain MNMs raises concerns regarding their use in facemasks. Limited research has addressed the airborne shedding potential of MNMs as it relates to inhalation uptake. This work aimed to address this gap by investigating the potential airborne release of TiO_2_ from the outer surface of three general purpose face masks, under two sets of conditions: physically stable and physically agitated. The surface of the face masks was analyzed to determine the mass fraction of TiO_2_ and the composition of the particles detected. Particle shedding was quantified via particle counters, particle sizers, electron microscopy, and inductively-coupled-plasma mass spectrometry (ICP-MS). Compositional analysis showed that all tested masks contained different levels of Ti ranging from 80 to 4870 µg/g of mask, with TiO_2_ particles detected on the surface of the fibers. Particle shedding was observed only for two of the masks under agitation with low average concentrations of 130 and 520 #/cm^3^. Further analysis of the shed particles did not indicate the presence of TiO_2_ nanoparticles. Overall, these findings suggest that while TiO_2_ was present in the face masks, its airborne release under typical conditions may be unlikely. Although additional efforts are needed to characterize a wider range of facemasks and expand on these data, this study contributes to the understanding of inhalation uptake from MNMs shedding to inform future risk assessments.

**Supplementary Information:**

The online version contains supplementary material available at 10.1038/s41598-025-34482-6.

## Introduction

Due to their capabilities and the growing need for high quality personal protective equipment during the Covid-19 pandemic, nanomaterials have been incorporated into face masks to enhance their microbiocidal activity^1, 2, 3, 4, 5^ and are found either blended into polymeric fibers or are coated on the surface of the fibers. Among the most commonly used nanomaterials in face masks are nanoscale silver (Ag), nanoscale zinc oxide (ZnO), nanoscale copper oxide (CuO), and nanoscale titanium dioxide (TiO_2_)^6^. Due to their photocatalytic properties, TiO_2_ nanoparticles, which are the focus of this study, have been shown to exhibit microbiocidal activity when exposed to light, due to the generation of reactive oxygen species^7^. Discussions related to the mechanism of nanoparticle microbiocidal activities can be found in^8, 9, 10, 11, 12, 13, 14^.

The integration of these nanomaterials to face masks raises the concern of their release (shedding) during typical or extended wear. Such release may occur due to agitation (e.g. breathing, coughing or sneezing) or abrasion (e.g. donning, doffing or mishandling), posing a possible inhalation exposure risk to the wearer ^1, 15, 16^. However, this remains poorly understood and characterized. Multiple studies have investigated and discussed the toxicity of nanoparticles^1, 11, 12, 15, 17, 18, 19, 20, 21, 22, 23, 24^, Shi et al.^25^ Wijnhoven et al. ^26^which showed that inhalation of such particles may cause adverse health effects, such as inflammation, oxidative stress, and lung damage. Therefore, it is important to assess the potential release of nanomaterials from these face masks as they inform potential inhalation exposure.

Most previous studies have evaluated the release of microplastics and nanoplastics from face masks, rather than metal-containing particles. Those studies have reported mixed results. A recent review by Kisielinski et al.^19^ compiled the results of multiple studies investigating the release of fibers, fiber components and chemical compounds from face masks, with many of the reviewed studies performed via leaching^20, 27, 28^, representing an upper limit for particle release. The authors report a significant portion of microplastics and nanoplastics to be potentially released via leaching, with N95-certified products. The released fraction was consistently higher compared to surgical face masks, largely attributed to the higher number of layers associated with N95 products. Only a few studies have investigated microplastics and nanoplastics in breathing-like apparatuses^20, 29^ to simulate direct inhalation exposure. Li et al.^29^ investigated particle release in air from different types of face masks under constant airflow, in an environment with no contamination control for a duration of 2 – 720 h. The authors observed an increase in the fiber released count with time and disinfection processes, except for N95 masks, which in general, showed lower fiber release. Further analysis using Raman spectroscopy showed that the particles collected consisted of 12% silica and 42% confirmed microplastics with size ranging from 20–100 µm. Meier et al. 2022^20^ investigated the release of fibers from face masks over 8 h using a Sheffield Head, with multiple donning and doffing, and compared their findings to the fraction released in liquid-based extractions. The authors found that under sinusoidal airflow, the fibers released from all masks were low, ranging between 6 to 24 fibres/g of mask. The number of fibers released slightly increased by a factor of 1.5 to 12.5 for different mask types when exposed to mechanical stress from donning and doffing. The number of fibers released using the breathing apparatus represented about 0.1 to 1.1% the fraction of liquid-based extractions for all masks tested.

The above-mentioned studies mainly focused on the detection of volatile organic compounds and microplastics/nanoplastics released from face masks. However, limited research has addressed the release of MNMs from face masks, which is crucial for assessing potential inhalation risks of metal or metal oxide nanoparticles. Due to the presence of these additive materials, detection of MNMs requires different characterization techniques not typically used for micro/nanoplastics. Bussan et al.^30^ investigated multiple surgical and K95 face masks for release of trace elements via leaching and from airflow under vacuum. While most face masks tested were found not to contain trace elements (i.e. were below the method detection limit), a few face masks were shown to contain traces of copper, antimony, zinc, and lead, where the latter (Pb) leached to approximately 60% after 6 h. In a comprehensive study, Verleysen et al.^16^ demonstrated, via scanning transmission electron microscopy coupled with energy dispersive X-ray (STEM-EDX) and inductively coupled plasma optical emission spectroscopy (ICP-OES), the presence of TiO_2_ from various face masks tested, with contents ranging from 17 to 4394 µg per mask available at the surface of the fibers, and median particle sizes ranging from 89 to 189 nm. A follow-up study by Montalvo et al.^31^ investigated the release of Ag-based biocides and TiO_2_ particles via leaching in artificial sweat. While Ag was released in amounts up to 36% of available Ag on the masks, TiO_2_ was found in minimal amounts (0.35% of available Ti on the face masks) in the released fraction. Similarly, Meier et al.^20^, Suwanroek et al.^32^ and Pollard et al.^33^ evaluated the leaching of multiple metals and metal oxides in deionized water, detergent and artificial saliva, which were found to vary with mask and solution. These leached amounts represent a worst-case scenario for exposure estimates and may not be realistic for estimating the potential for inhalation.

To our knowledge, there are currently no published studies reporting direct measurements of airborne shedding of component MNMs from commercial mask products. Such data are crucial to support risk assessments with more realistic inhalation uptake and exposure estimates, as highlighted by the recent review of Everaert et al.^15^. Some attempts to address this issue have been reported^34^ but with inconclusive results, due to a large variability in the observations.

In this work, we performed an exploratory assessment of potential airborne particle shedding from three different face mask products, all claimed to contain TiO_2_. We first characterized and quantified nanomaterials and Ti content for each mask, then evaluated airborne particle shedding using a custom-built setup, under controlled conditions and constant flow. Shed particles were analyzed based on their number, size and composition.

## Results

### Surface characterization and compositional analysis

**Fig. 1:**
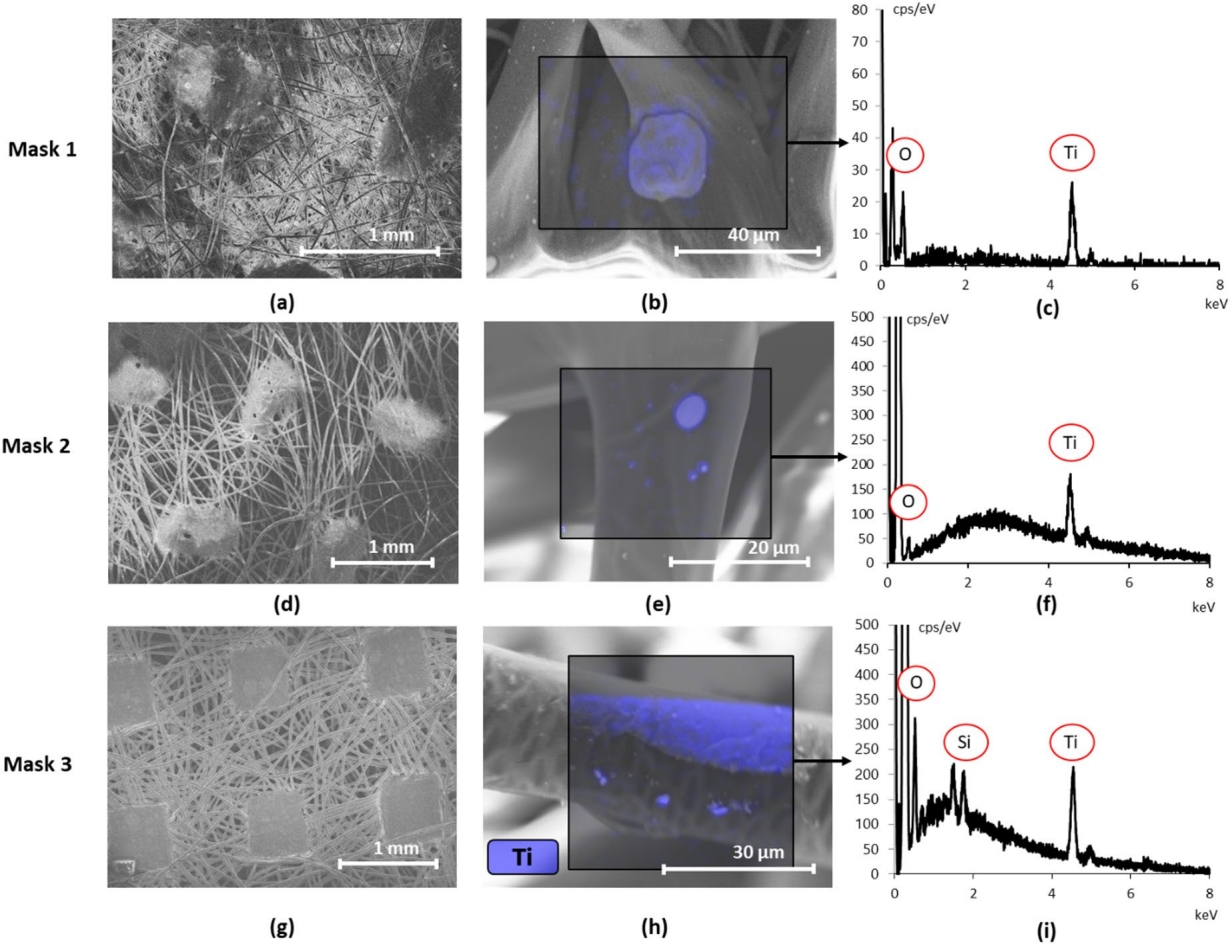
SEM images of the outer layer of (**a**) Mask 1, (**d**) Mask 2 and (**g**) Mask 3 with a higher magnification and EDX elemental map for Ti shown in (**b**), (**e**) and (**h**) respectively. The corresponding sum spectrum of the EDX elemental mapping is shown in (**c**), (**f**) and (**i**) for Mask 1, Mask 2 and Mask 3 respectively.

Following the methodology proposed in Section "Surface characterization", all layers of each face mask were imaged with SEM (with the exception of layers 3–5 of Mask 3), with EDX performed on the detected particles. Particles were observed on all outer layers of the mask as well as the 1^st^ middle layer of Mask 3 and the inner layer of Mask 2. Figure 1, panels a, d and g, present low magnification images of the outer layers of Mask 1, Mask 2 and Mask 3, respectively. Higher magnification images are shown in Fig. 1, panels b, e, and h with the elemental Ti map overlaid, to illustrate the Ti distribution within the region of interest (ROI), outlined by a black box. Figure 1, panels c, f and i, show the corresponding EDX sum spectra, representing the sum of all individual pixel spectra in the ROI.

All layers displayed in Fig. 1 show a similar fiber network, characteristic of a non-woven spunbonded layer with approximate fiber dimeters of 16 µm, 20 µm and 30 µm for Mask 1, Mask 2 and Mask 3 respectively. Overall, the EDX analysis demonstrates that most particles on the outer layers contained Ti and O, with some particles containing Ag and Si occasionally found on the surface of Mask 2 and Mask 3 respectively.

**Fig. 2:**
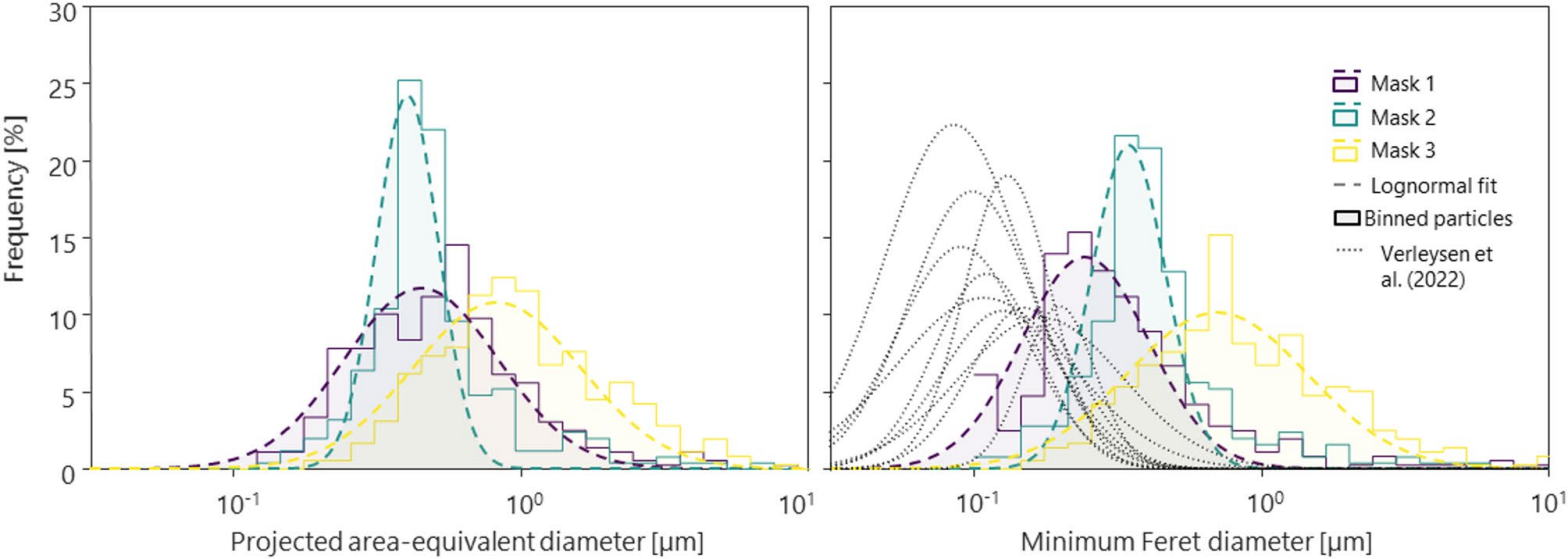
Particle size distribution for Mask 1, Mask 2 and Mask 3 as a function of the projected area-equivalent diameter (left panel) and minimum Feret diameter (right panel), with corresponding lognormal distribution fit to the data. These results are compared to size distributions of agglomerated TiO_2_ particles obtained by Verleysen et al.^16^ for 10 face masks.

The particle size distributions for the outer layers of Mask 1, Mask 2 and Mask 3, based on a total of 358, 250 and 355 particles, respectively, are shown in Fig. 2 as a function of the projected area-equivalent diameter. A lognormal distribution was fitted to each dataset. Masks 1 and Mask 3 exhibited similar distributions, although larger particles were detected for Mask 3 (modes: 0.52 µm and 0.79 µm for Mask 1 and Mask 3 respectively), likely due to the presence of a Ti-coated layer on the fibers (e.g. Fig. 1h). This observation supports Mask 3 manufacturer’s claim for the presence of a TiO_2_ coating on the outer layer. However, since no additional details were provided regarding the coating material or integration process, further conclusions could not be drawn. While Mask 1 and Mask 3 displayed a broad range of particle sizes, Mask 2 showed a narrower size distribution, with the mode of the distribution around 0.37 µm and most particles below 1 µm.

The particle size distributions, expressed as a function of the minimum Feret diameter, shown in the right panel of Fig. 2, are compared to the findings of Verleysen et al.^16^. In their study, Verleysen et al. analyzed 12 samples using a high-angle annular-dark-field scanning transmission electron microscope (HAADF-STEM) to image agglomerates within the fiber cross-section. Size distributions reported by Verleysen et al. from the outer layer of 10 of the 12 samples were fitted using a lognormal distribution and compared to our findings.

In general, Verleysen et al. reported narrower particles size distributions for most samples with modes ranging between 0.1 to 0.3 µm. This difference likely stems from the different imaging techniques and sample preparation. In our study, a small area of the outer layer was imaged by SEM, whereas Verleysen et al. examined a thin cross-sectional area by HAADF-STEM. In our study, the resolution was limited by the pixel size (10 to 50 nm depending on the magnification), therefore excluding particles below ~ 100 nm due to the large uncertainties associated with their measurement. The morphology of particles was not further assessed to distinguish between single particles or agglomerates, as this was beyond the scope of this study.

Analyzing the inner layers of the face masks (supplemental information Fig. S2), particles were only clearly detected on Mask 2. EDX performed on the particles detected, did not show the presence of TiO_2_ in Mask 2. Consequently, the subsequent analysis mainly focused on the outer layer of the face masks.

### Total Ti content

Analysis of the digested mask samples by ICP-MS revealed the presence of Ti in all face masks, with varying quantities detected. The mass fractions of Ti per g of face mask is provided in Table 1 for all replicates as an average of 3–5 subsamples with corresponding standard deviation (SD). Detailed results for all replicates are provided in Table S1 in the supplemental information. Mask 1 was found to have the highest content of Ti per g of mask, and hence was considered to be a good candidate for the subsequent shedding experiments. However, it should be noted that the mass fraction of Ti in this mask, is associated with high relative standard deviation (RSD), representing the ratio of the standard deviation to the mean, between subsamples of the same replicate, ranging between 19 and 43%. This suggests that the TiO_2_ coating of the mask fiber is not homogeneous although the RSD of total mass fraction of Ti among the replicates of mask 1 is within 9%. On the other hand, the TiO_2_ coating on mask 2 seemed to be homogeneous with RSD ranging between 3 and 5%, but noticeable differences were observed among the mask replicates with RSD of 16%. Mask 3 contained the lowest mass fraction of Ti and a large RSD was observed within the studied subsamples, between 7 and 35% as well as among the mask replicates with RSD of 38%. Ti was found in Control 1, with similar mass fractions to Mask 3.

**Table 1:** Weighted averages of Ti mass fractions (µg/g of mask) from ICP-MS over the replicates and the associated uncertainties (k = 1) before (Original) and after 48 h of agitation in ethanol (After agitation). The portion released provides an estimate of the fraction of particles released after agitation in liquid.

		Original		After agitation		
Mask	Replicate	Repeats, *n*	Ti Mass fraction ± SD [µg/g]	Repeats, *n*	Ti Mass fraction ± SD [µg/g]	Portion released
Mask 1	3	5	4290 ± 550	3	4290 ± 690	< 1%
Mask 2	3	3	1940 ± 40	3	1680 ± 65	< 1%
Mask 3	3	4	95 ± 5	3	43 ± 7	~ 53%
Control 1	3	3	192 ± 25	3	LOD	-
Control 2	1	3	LOD	-	-	-

LOD: below limit of detection < 0.464 µg/g, SD: standard deviation.

*n*: Number of subsamples for each replicate.

### Particle release in liquid

**Fig. 3:**
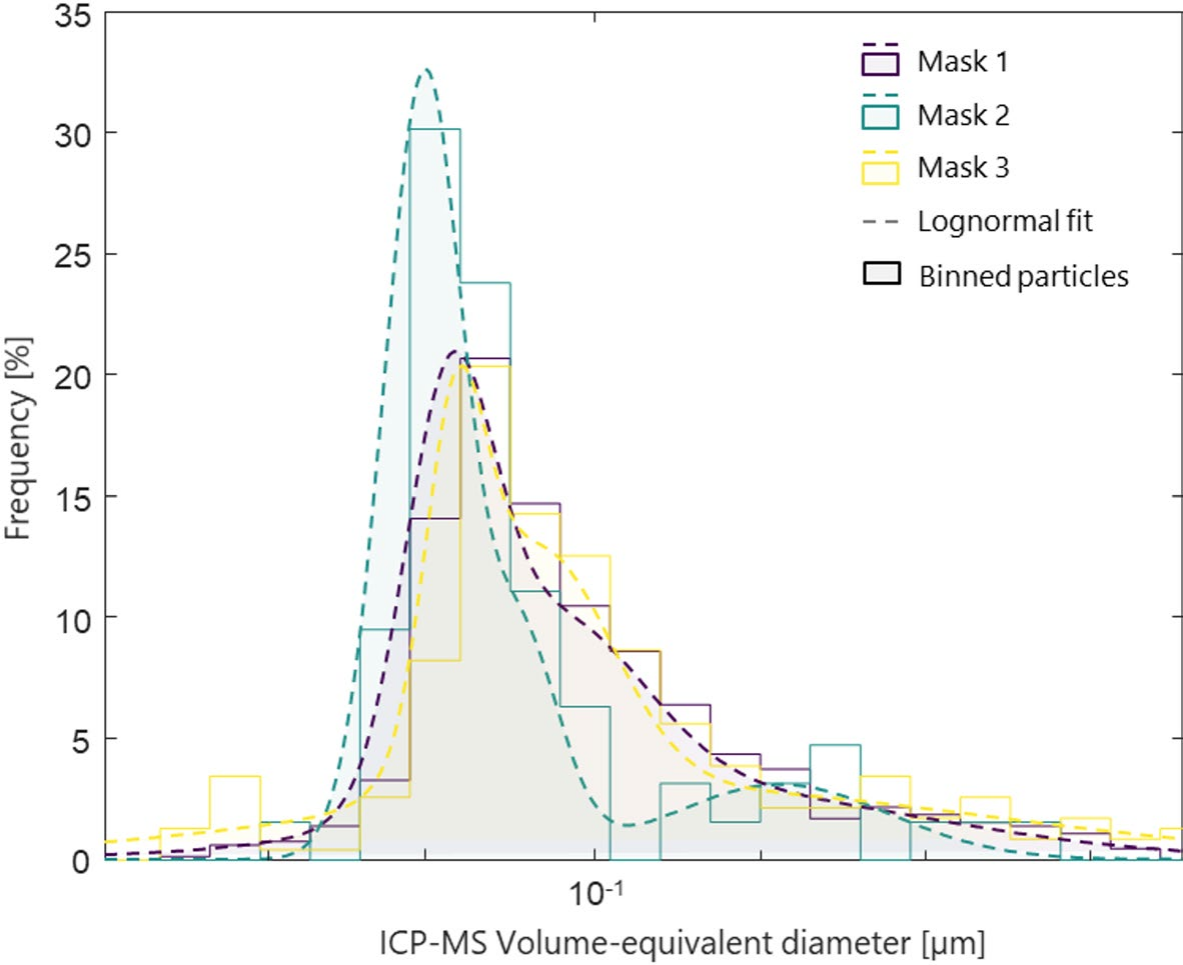
Size distribution of TiO_2_ nanomaterials in ethanol leachate of agitated masks detected by single particle ICP-MS in Mask 1, Mask 2 and Mask 3.

Figure 3 presents the particle size distribution of the released TiO_2_ particles in liquid, after 48 h of agitation in ethanol, as analyzed by SP-ICP-MS for Mask 1, Mask 2 and Mask 3. A visual inspection of the supernatant revealed a discernible amount of fiber released from Mask 3, compared to Mask 1 and Mask 2. Analysis of the supernatants showed < 100 particles detected per sampling time for Mask 2, as opposed to ~ 200 and ~ 600 particles detected for Mask 1 and Mask 3 respectively. Interestingly, all masks exhibited similar particle size distributions, with modes around ~ 60 nm. While Mask 2 showed a slightly narrower range, the small number of particles detected introduced higher uncertainties in the size estimation. The size distributions were fitted using a multivariate lognormal distribution to account for the larger particle sizes detected. The small range of particle sizes was not observed with the SEM surface analysis due to the limited image resolutions. However, it is important to note that projected area-equivalent diameters obtained by image analysis are not directly comparable to volume-equivalent diameters obtained by SP-ICP-MS. A negligible number of particles were detected in the control samples and therefore are not shown in Fig. 3.

Digestion and analysis of the agitated samples in ethanol allows for a direct comparison with the original intact sample, providing better insights on the shedding potential of each face mask. Table 1 provides a comparison of the Ti mass fraction measured from digested samples of Mask 1, Mask 2 and Mask 3 before and after agitation. Only a small fraction of Ti (< 1%) could be displaced from Mask 1 and Mask 2  by leaching, whereas about 53% of the Ti was found to be displaced from Mask 3 due to leaching. However, a high uncertainty is associated with the released portion due to the variability in measured Ti fraction between subsamples.

Since liquid extraction would represent an upper limit for particle release^20, 27, 28^, these results imply a low airborne shedding potential of TiO_2_.

### In-situ shedding during physical agitation

**Fig. 4:**
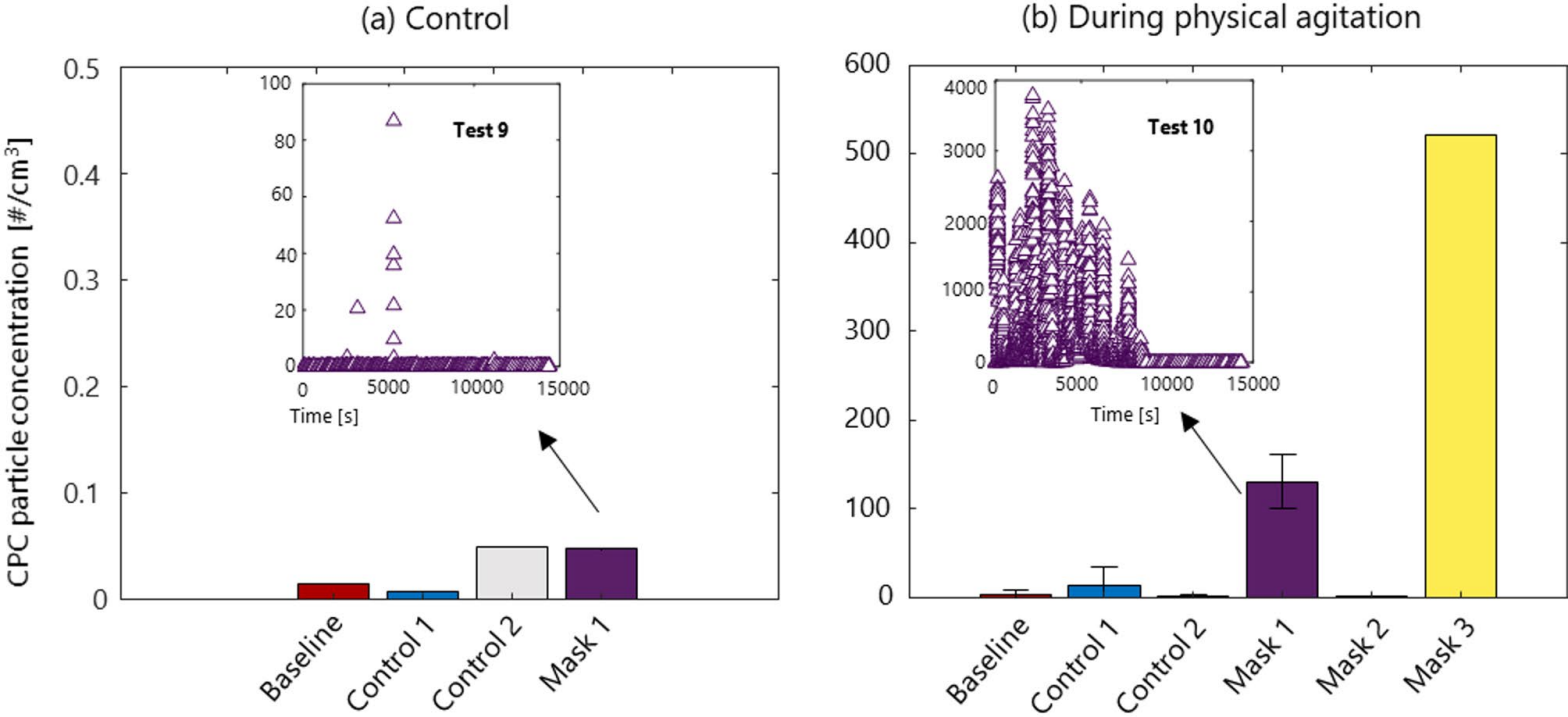
Particle number concentration obtained from average measurements of each test performed in Table 3 for both control face masks and all three face masks tested (**a**) without and (**b**) with inline agitation (vibration). The inset time series show example test runs for each case.

The particle number concentration obtained by the downstream CPC for each test, as summarized in Table 3, was averaged over the test duration and is shown, in Fig. 4panel a and panel b without and with inline agitation respectively. Results are presented as standalone single tests or as an average of 2 to 4 replicate tests (± standard deviation). As illustrated in the examples provided for Test 9 and Test 10, shedding was not observed continuously. Instead, an intermittent particle release was observed with concentrations varying between tests.

**Fig. 5:**
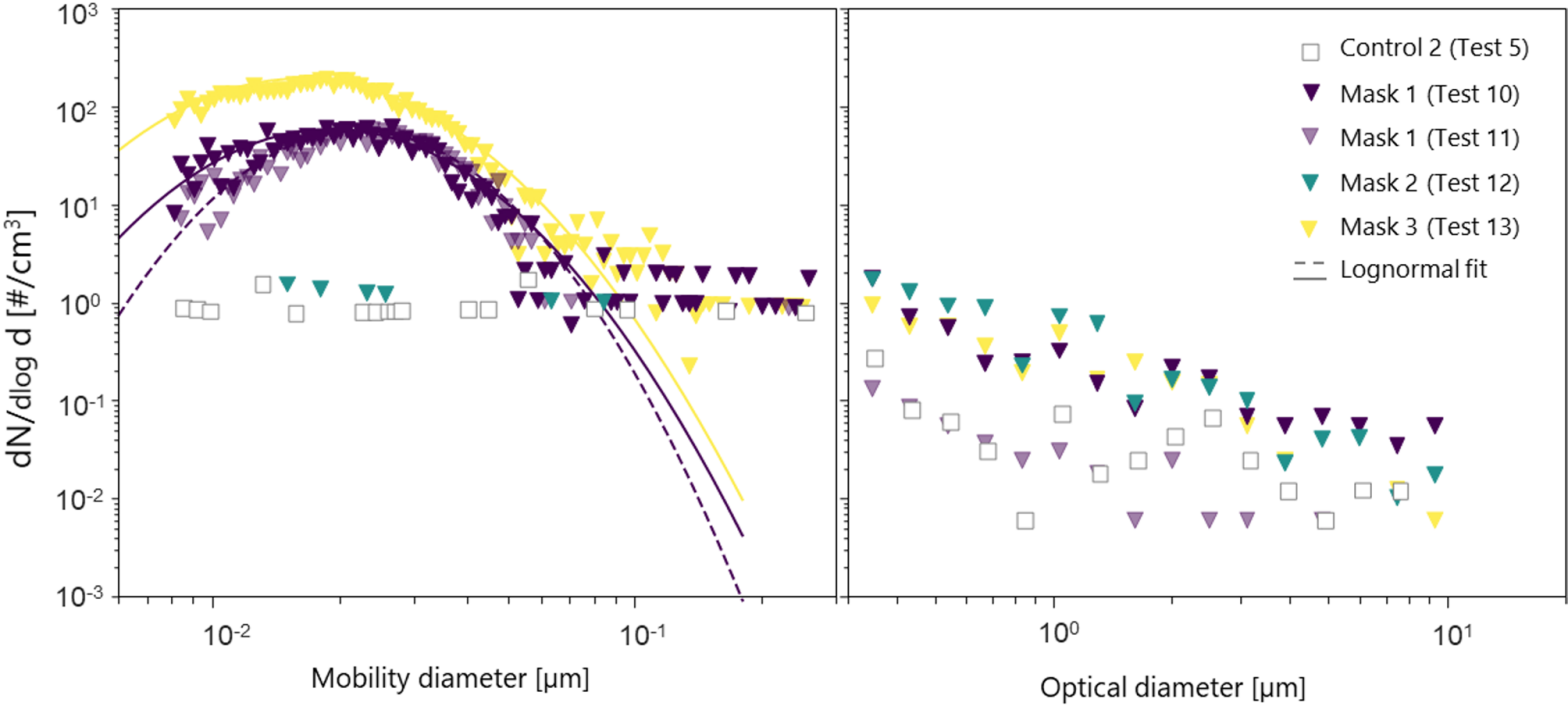
Number size distribution in time for Mask 1, Mask 2 and Mask 3 tested under constant flow with inline agitation, as measured by SMPS (left panel) and OPS (right panel). SMPS size distributions were fitted using a lognormal distribution. Flat points in the left panel correspond to the detection limit of the CPC.

Minimal shedding was observed (< 0.1 #/cm^3^) under continuous airflow with no inline agitation, for all face masks tested. With the addition of inline agitation, a slight increase in particle count was noted for the Control 1, with a mean of 13 #/cm^3^, while Control 2 showed negligible particle release about 1 #/cm^3^. The highest concentrations were found for Mask 1 with a mean and standard deviation of 130 ± 30 #/cm^3^ and for Mask 3 with a concentration of 520 #/cm^3^ indicating a significant increase compared to the control conditions. Size distributions for Mask 1, Mask 2, Mask 3 and Control 2 under constant flow with inline agitation, measured simultaneously by the SMPS and OPC, are shown in Fig. 5 integrated over the entire test period. These results again highlight the sporadic nature of the shedding observed. Most particles were found below 50 nm, with varying particle concentrations in time. Notably, the number concentrations for Mask 2 were found comparable to those of Control 2.

For all tests, the larger particle counts and size distributions, as measured by the OPS, remained consistently below 1 #/cm^3^ and were therefore considered negligible. These results are consistent with the size distributions measured in the particle release experiments conducted in liquid, despite the low particle counts observed in both datasets. The low particle counts obtained across all experiments did not allow for a reliable estimation of the polydispersity index (PDI), which reflects the spread of the particle size distribution. This parameter is important for exposure models, as it captures variability in particle sizes that can influence particle transport and deposition. Insufficient particle counts would make PDI estimates unreliable for these applications.

Characterization of the particles collected on PVC filters for Mask 1 with inline agitation using ICP-MS, after the complete digestion of the filter and particles, did not show the presence of Ti in any of the samples above the limit of detection.

**Fig. 6:**
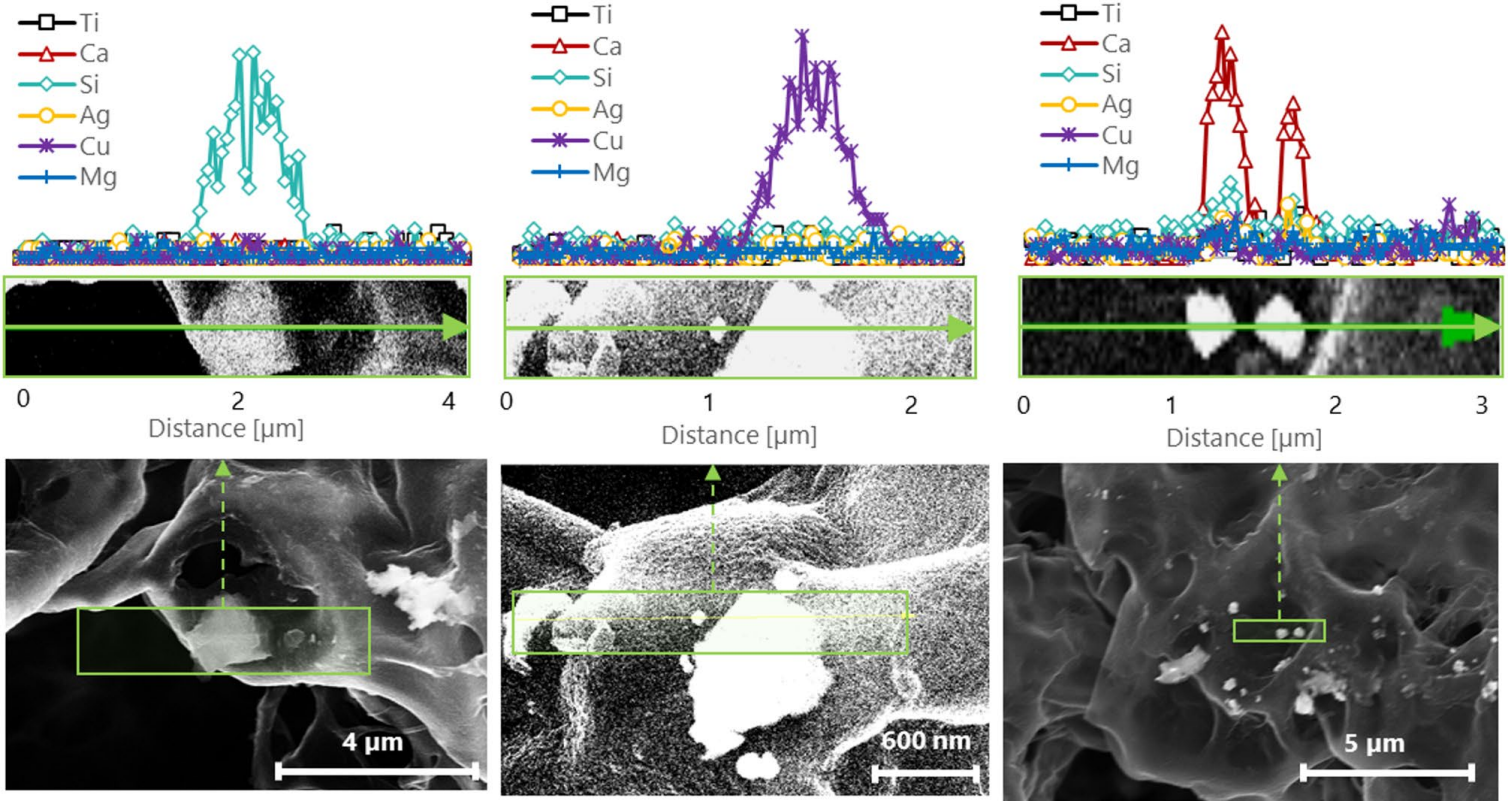
STEM images and EDX line spectrum for three representative particles observed on the PVC filter, collected from Mask 1 under constant flow and inline agitation (Test 10). Particles were found to contain mostly Ca, Cu or Si, while no TiO_2_ particles were detected.

PVC filters collected from the 4-h tests performed with Mask 1 without and with inline agitation (Test 9 and Test 10 respectively) were analyzed with STEM-EDX to investigate the composition of the particles collected. Consistent with the average particle number concentrations, an inspection of the filter surface revealed no detectable particles without agitation, whereas a limited number of particles (~ 20) were observed with the addition of inline agitation. Figure 6 shows images of three representative particles observed on the PVC filter from Test 10. EDX was performed across the ROI, through the particles and its surroundings, to provide a profile of the elements detected. The EDX analysis did not detect the presence of TiO_2_ particles. Most of the particles analyzed contained Si, Cu or Ca with two particles showing minimal levels of Mg. Of these elements detected, only a small number of Si-containing particles were found on the surface of the face masks. As no particles containing Ca or Cu were previously detected on the surface of the face mask, it remains unclear whether such particles originate from the mask itself or result from contamination during handling. The face masks were obtained as commercially available products, and the details of the nanoparticle integration process were not disclosed by the manufacturers. It is possible that the contact between the vibration motors and the mask may have caused particle release via physical abrasion, although such abrasion may still be representative of some real-world scenarios such as mask donning and mishandling. However, abrasion is expected to produce micron-sized particles, of which negligible concentrations were observed.

Although, a limited number of detected particles were analyzed, the results, in conjunction with the total filter characterization performed using ICP-MS, suggest that minimal shedding would occur, even under extreme and unrealistic agitation conditions. The shedding potential is expected to decrease with the number of layers of the face mask, assuming the particles are only located on its most outer layer.

## Discussion

Due to the varying nature of the airborne shedding observed, definitive conclusions could not be drawn based on the three face masks and repeats performed. Analyzing the face masks selected, the highest and lowest levels of Ti were found for Mask 1 and Mask 3 respectively, with Mask 3 showing the highest number of TiO_2_ particles released in liquid (representing ~ 50% of the mask content). Mask 2 showed high levels of Ti but a negligible number of TiO_2_ particles released in liquid. Airborne shedding was the highest for Mask 3, with Mask 2 showing a negligible number of particles released. These results suggest that, although nanoparticles were detected on the surface of the face mask fibers, the released fraction, if any, would not be sufficient for comprehensive characterization.

No TiO_2_ was detected on the apparatus’ airborne collection filters, indicating no or negligible release of TiO_2_ from the face masks, so it was not considered relevant nor appropriate to use the measured particle number air concentrations to estimate inhalation exposure for TiO_2_. For context, the highest measured particle number concentration of 520 particles/cm^3^, observed only for one of the masks (mask 3) tested under unrealistic agitation conditions, is nearly an order of magnitude lower than ultrafine particle (aerodynamic diameter of 100 nm or less) number concentrations measured in North American reference rural ambient air (approx. 5000 particles/cm^3^) and orders of magnitude lower than North American urban air (typically ranging 10,000 to 15,000 particles/cm^3^)^35^. Given the low numbers of mixed-composition particles in the ultrafine size range being released from the mask under unrealistic agitation, and the non-detect of Ti or TiO_2_, the number concentration of released particles and the potential for inhalation exposure is considered negligible.

Our results align with the limited studies in the literature. Verleysen et al.^16^ investigated the content of 12 face masks for TiO_2_ and Ag nanomaterials, using a similar approach to the one described in our study. The authors combined ICP-OES analysis to quantify the total amount of TiO_2_ (ranging from 791 to 152,345 µg per mask) and TEM analysis to determine the particle fraction on the fiber surface (~ 2 to 4% of the particles detected). They reported 17 to 4394 µg per mask of TiO_2_ available at the surface of the face masks. Our findings are not directly comparable with the study of Verleysen et al. considering the different techniques used for the mask characterization. In this study, the titanium mass fraction analyzed by ICP-MS was quantified only in the outer layer, considering the SEM–EDX analysis performed prior. However, adopting a similar analysis, where only 2–4% of the particles are available on the fiber surface and all Ti is present as TiO_2_, we expect TiO_2_ levels of approximately 1002 µg, 330 µg and 53 µg for each of Mask 1, Mask 2, and Mask 3, respectively (based on the weights reported in Table 2), which is within the range of levels reported by^16^.

**Table 2:** Description of selected Mask 1, 2 and 3. Layer 1 refers to the material facing outward from the wearer, while the highest layer refers to the layer in contact with the wearer’s face.

Face mask	Type	Weight ± SD [g]*	Layers
Mask 1	General purpose face masks Disposable	3.42 ± 0.02	Layer 1: SB non-woven PP (outermost)
			Layer 2: MB non-woven PP
			Layer 3: MB non-woven PP
			Layer 4: SB non-woven PP (innermost)
Mask 2	General purpose face masks Disposable	3.09 ± 0.02	Layer 1: SB non-woven PP
			Layer 2: MB non-woven PP
			Layer 3: SB non-woven PP
Mask 3	General purpose face masks Reusable	10.58 ± 0.04	Layer 1: Not disclosed
			Layer 2: Activated carbon
			Layer 3: Electrostatic mesh
			Layer 4: MB non-woven PP Layer 5: Not disclosed
			Layer 6: Cotton layer
Control 1	Surgical mask Disposable	2.95 ± 0.02	Layer 1: SB non-woven PP
			Layer 2: MB non-woven PP
			Layer 3: SB non-woven PP
Control 2	General purpose face masks Reusable	2.39 ± 0.02	Layer: Nanospun non-woven PVDF, PLGA/PAN

SB: Spunbond, MB: Meltblown, PP: polypropylene, PVDF: Polyvinylidene fluoride, PLGA: poly(lactic-co-glycolic acid), PAN: Polyacrylonitrile.

*Reported weight is based on measurements of three replicates of each face mask.

Montalvo et al.^31^ further investigated the release of Ag-based biocides and TiO_2_ particles from the same face masks analyzed in Verleysen et al.^16^, via leaching in artificial sweat. Out of 10 face masks analyzed via ICP-MS, only 2 face masks showed detectable Ag levels in the leachates, while Ti was detected for 1 face mask only. The Ti mass fraction leached (5.5–8.4 µg/g for two replicates) is comparable to our findings with levels ranging from ~ 1 to 37 µg/g in the supernatant of samples from Mask 1 and Mask 3 respectively.

To our knowledge, this study is the first to report direct airborne shedding from face masks providing released particle concentrations and composition and therefore cannot be directly compared to the literature. However, Mast et al.^34^ reported an attempt to quantify airborne shedding from three replicates of three face masks confirmed to contain TiO_2_ and Ag, on a Sheffield headform and breathing apparatus, following the guidelines outlines in the EN 149:2001 + A1:2009 standard testing procedure for respiratory protective devices. Although particles were detected on all samples, large variations were found between replicates and samples. When compared to experiments performed with a control face mask, no TiO_2_ above the baseline measurements were detected. These conclusions are similar to our findings for airborne shedding where, although particles are observed, Ti levels were below our detection limits, for all techniques employed.

Conclusions from our work and the literature, seems to indicate that nanomaterial shedding or resuspension from the different types of face masks, would not likely occur under the tested conditions. Specifically, our analysis performed with NaCl loaded Control 2 face masks during the experimental validation (see supplemental information Fig. S3) shows insignificant resuspension (less than 0.6 particles resuspended per million particles in the mask). This is in agreement with multiple studies investigating the occurrence and mechanism of submicron (< 1 µm) and supermicron (> 1 µm) particle shedding from textiles and fibrous materials. Particles depositing onto filter material will adhere by van der Waals forces, electrostatic forces or capillary forces^36^, which depends on the filter material, particle size and humidity levels. These particles can potentially shed if external forces are applied, such as high airflow or mechanical agitation, exceeding the adhesion force^37^.

Qian et al.^36^ investigated the resuspension of polystyrene latex spheres, sodium chloride, corn oil and dust (ranging from 0.6–5.1 µm in aerodynamic diameter) from three types of filters – a respirator cartridge (glass fiber filter) and two half mask respirators (polypropylene filter)—by subjecting each sample to a maximum air velocity of 500 cm/s. The particles were detected downstream of the filter material using a combination of an Amherst Process Instruments (API) Aerosizer and a photometer. At the maximum air velocity tested, particle resuspension was found to be negligible (~ 0.3%) for particles below 1 µm and increased to 17% for the largest particles tested. The authors also report a minimum entrainment velocity of 130 cm/s to detect particles below 1 µm. Similar findings were reported by Qian et al.^38^ when comparing shedding of inert particles with bacterial aerosol particles from N95 respirators, in which resuspension up to 0.025% for particles of 0.8 µm at 300 cm/s was observed. Fisher et al.^39^ report a maximum of 0.21% MS2 bacteriophage resuspension, as a droplet and droplet nuclei, under cyclic flow.

Other studies have investigated particle release from filter material under conditions beyond airflow such as material stretching or mishandling (drop from different heights), and report similar findings with only a negligible fraction of particle released^40, 41^.

The limited evidence from this study, together with resuspension studies reported in the literature, further indicates that particle release from face masks, and therefore inhalation exposure to TiO_2_, is likely negligible. However, it is crucial to expand on these findings by testing a broader range of face masks containing different MNMs with multiple repeats considering the variability in the results observed.

## Methods

### Face mask selection

A survey of commercially available face masks was performed to identify specific masks claiming to either provide “antimicrobial” properties, provide “self-cleaning” capabilities or simply contain nanomaterials. Most commercially available masks surveyed claimed to contain Ag, with a small number of masks containing TiO_2_, ZnO, SiO or CuO. Based on this survey and availability, we selected three masks claiming to contain TiO_2_, referred to herein as Mask 1, Mask 2 and Mask 3, all of which were general purpose face masks available to consumers. The breakdown of the layers in these masks is provided in Table 2, with images of the masks available in the supplemental information (Fig. S1). For sample selection, we ensured all face masks consisted of different number of layers.

Two additional face masks with no claims of TiO_2_ presence, were selected as control samples. The first control face mask (Control 1) represents a generic 3-layer surgical face mask while the second face mask (Control 2) represents a 1-layer non-woven nanofiber mask. The selected face masks were analyzed to determine the content, particle size, composition and location of TiO_2_ particles in the masks, as well as their shedding potential.

### Face mask characterization

#### Surface characterization

One sample was selected from each face mask. Each layer of the three face masks (with the expectation of layers 3–5 of Mask 3) were analyzed using scanning electron microscopy (SEM) and, where particles were observed, further examined with energy dispersive X-ray (EDX). SEM–EDX was used as an initial qualitative assessment to verify the presence of TiO_2_ particles on the surface of the face masks and estimate the sizes of the detected particles. This method was not intended as a quantitative method for determining the amount of Ti present on each face mask. For this analysis, the layers of each face mask were separated and cut into 100 mm × 100 mm samples, which were imaged on both the outer and inner surfaces.

To minimize charging effects due to a charge accumulation from the electron beam, carbon was sputter-coated on each sample prior to SEM, resulting in a 10–25 nm coating. Samples were first imaged using high resolution SEM (S-4800, Hitachi Ltd., Japan) to obtain large scale images ranging from 3.6 × 2.5 mm to 84 × 60 µm. For more detailed characterization, ultrahigh resolution SEM (S-5500 Hitachi Ltd., Japan), coupled with EDX analysis, was performed on smaller sample areas ranging from 84 × 60 µm to 50 × 35 µm. Where particles were observed, EDX elemental mapping was performed simultaneously to determine the elemental distribution across the scanned area.

Image analysis, using ImageJ software (National Institutes of Health, USA), was performed on several SEM images to obtain a size distribution of observed particles available on the surface of each face mask. For this purpose, most particles were outlined with an ellipsoid, with the exception of large irregular particles observed, which were outlined following their shape. Size distributions were obtained based on minimum Feret diameter and the projected area-equivalent diameter calculated from the projected area of the particle.

#### *Analysis of dissolved and particulate TiO*_*2*_* by ICP-MS*

For the total metal content, samples were acid-digested as described elsewhere^42^. Briefly, 10 mg of the face mask was digested in 2 mL of concentrated nitric acid (HNO_3_) and 4 mL concentrated sulfuric acid (H_2_SO_4_) in a closed microwave system (Multiwave 7000, Anton Parr GmbH, Austria) using the following digestion program: 15 min ramp to 280 °C followed by a hold period of 60 min at 280 °C. Each face mask sample was digested in triplicate, with 3–5 subsamples (10 mg portion) for each replicate, and each digestion cycle contained at least one method blank and one certified reference material (SRM 1898 – Titanium dioxide nanomaterial).

Digested samples were diluted to a final HNO_3_ acid concentration of 2% and analyzed using an Agilent 8900 ICM-MS/MS (Agilent Technologies, Santa Clara, CA, USA) introduced using an Agilent SPS 4 autosampler with a cover (Agilent Technologies, Santa Clara, CA, USA). Additional details about the instrument settings are provided in the section S2 of the supplemental information. To verify the accuracy of the digestion method, at least one replicate of NIST SRM 1898 (Titanium dioxide nanomaterial) was included in each digestion cycle. The recoveries for Ti mass fraction in this SRM were withing acceptable range (92 ± 9%, *n* = 10). Possible sources of Ti contamination were monitored through method blanks, which were included in each digestion cycle and treated as samples through the entire analysis. No Ti contamination was detected. The method limit of detection was calculated as 3*σ* of blank samples (*n* = 7) with nominal dilution factor of 5000 and equal to 0.464 µg/g.

For characterization of particle shedding potential by leaching, three accurately weighed subsamples from one replicate of each face mask (10 mg) were placed in a vial containing 30 mL of ethanol. The samples were agitated on a rotary shaker (Roto-Shake Genie, Scientific industries Inc., USA) for 48 h. The supernatant was collected from each sample, evaporated to near dryness, reconstituted in 10 mL of MilliQ water and analyzed by ICP-MS in single particle mode (SP-ICP-MS).

The remaining mask samples (after shaking) were dried and digested for analysis of their remaining Ti content, following the methodology outlined above.

### Particle shedding

#### Experimental setup

Particle shedding was assessed using a modified version of the custom-built system^43, 44^ previously employed for particle number-based filtration efficiency and pressure resistance measurements of various face masks^45, 46^. The experimental setup consisted of a testing chamber; a pair of condensation particle counters (CPCs Model 3752 and Model 3750, TSI Inc., USA), placed one before and one after the testing chamber; a scanning mobility particle sizer (SMPS), which consists of an electrostatic classifier (Model 3082, TSI Inc., USA), an aerosol neutralizer (Model 3088, TSI Inc., USA) and a CPC (Model 3752 and Model 3750, TSI Inc., USA), placed after the testing chamber; and a vacuum pump which drew HEPA filtered air through the system. The mask samples were sealed to a mounting plate. The plate and samples were mounted in the testing chamber with the outer side of the mask facing the incoming air to mimic the direction of air flow from inhalation during mask use. The relative humidity and temperature were maintained at 40 ± 10% and 21 ± 2 ºC, respectively, in the chamber. Clean and humidified air, filtered through a set of HEPA filters, traversed the testing chamber. The upstream particles were sampled with the first CPC, while the particles downstream were sampled with the second CPC, SMPS and an optical particle sizer (OPS, Model 3330, TSI Inc., USA) accounting for a broad range of sizes from 5 nm to 10 µm. The flow rate was controlled via a mass flow controller (EL-Flow, Bronkhorst, Netherlands) and was maintained at 50 ± 5 L/min. Background measurements, without no mask on the mounting plate, were performed prior to each sample measurement day.

**Fig. 7:**
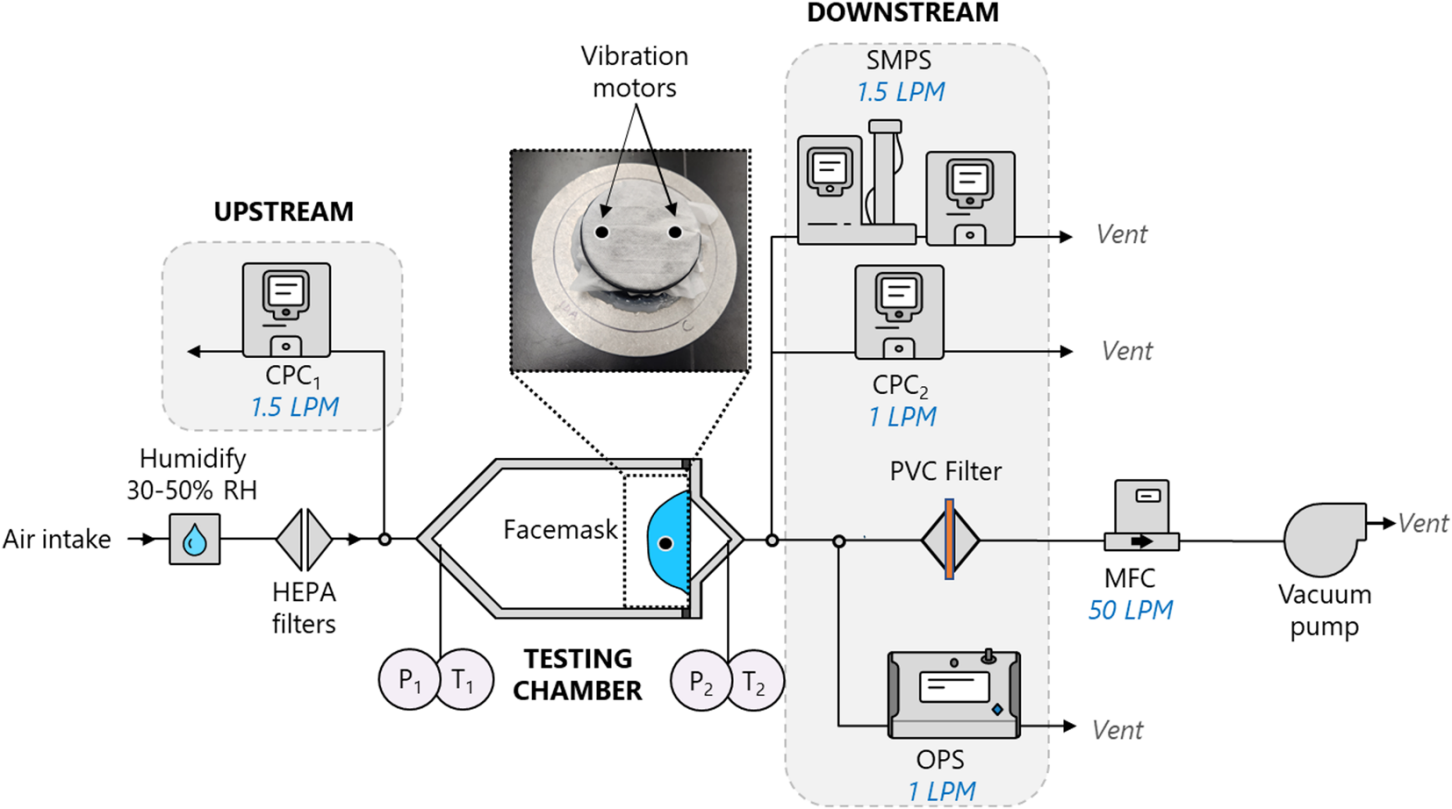
Experimental setup for particle release in air. The setup is based on a custom-built system used for particle filtration efficiency measurements but is modified to accommodate for particle detection downstream using a condensation particle counter (CPC), a scanning mobility particle sizer (SMPS), an optical particle sizer (OPS) and a filter sampler for all particle collection. MFC: mass flow controller, PVC: polyvinyl chloride.

Intensive inline mask agitation (vibration) of the sample was also implemented using two vibration motors (10 × 3 mm, stainless steel, 12,000 RPM) placed on the inner surface of the mask within the testing chamber, as shown in Fig. 7. Each vibration motor was placed onto the surface of the mask using magnets, ensuring a rapid connection and minimizing damage to the surface of the face mask. The vibration was controlled via a voltage adjuster allowing a vibration frequency of up to 200 Hz (cycles/sec).

This high agitation frequency does not represent the agitation of a face mask during typical breathing, where a normal breathing rate of 10–20 breaths per minutes corresponds to a breathing frequency of 0.16–0.33 Hz, but rather provides an upper limit for potential particle release. Such agitation could also be understood to represent the agitation that occurs in a person’s pocket or during other mishandling of a mask.

#### Procedure and particle collection

Unless otherwise stated, all tests were conducted using only the outer layer of each mask only, facing the incoming air, to minimize variability due to differences in the number of layers between masks. Prior to testing, the seams were cut to release the pleats and separate the mask layers.

At the beginning of each measurement day, a background or blank test was performed with and without vibration, providing a baseline to all instruments. Once the face mask was sealed in the testing chamber, humidified filtered air (~ 40% relative humidity) was passed at a constant flow of 50 L/min for 20 min to 4 h. All mask samples were then tested without further manipulation. Shed particles were continuously monitored using a CPC for small particle sizes below 3 µm, a SMPS (1 min scan) for particle sizes below 1 µm and an OPS (1 min collection) for sizing and counting from > 0.3 µm to 10 µm. The remaining particles were collected on Polyvinyl chloride (PVC) membrane filters, with a nominal pore size of 5 µm (Millipore Sigma, USA) for metal content analysis using ICP-MS (following the same digestion procedure outlined in Section "Analysis of dissolved and particulate TiO2 by ICP-MS") and/or STEM-EDX analysis. Although a large nominal pore size was used, the PVC filters demonstrate high collection efficiencies across a range of particle sizes^47^.

TEM grids collecting the shed particles were not assessed in this study due to the low particle concentration < 4% loading on the grids. The complete list of all tests performed is detailed in Table 3.

**Table 3:** List of experiments performed, detailing the different experimental conditions for each test.

Test	Face mask	Duration (min)	Vibration	Analysis
1	Control 1 (× 2)	20	No	-
2	Control 1 (× 2)	20	Yes	-
3	Control 1, 3 layers	20	Yes	-
4	Control 2 (× 2)	20	No	-
5	Control 2 (× 2)	20	Yes	-
6	Mask 1	20	No	ICP-MS
7	Mask 1 (× 2)	20	Yes	ICP-MS
8	Mask 1, 4 layers	20	Yes	ICP-MS
9	Mask 1	240	No	STEM-EDX
10	Mask 1	240	Yes	STEM-EDX
11	Mask 1	60	Yes	SP-ICP-MS
12	Mask 2	20	Yes	SP-ICP-MS
13	Mask 3	20	Yes	SP-ICP-MS

The air upstream was monitored with a CPC to ensure no room air contamination is present in the system. The apparatus including the counting and sizing instruments, was validated using a sodium chloride loaded Control 2 face mask (detailed in section S4 of the supplemental information) and shown to effectively capture a small fraction of the loaded particles.

## Conclusions

Three face masks claimed to contain TiO_2_, were characterized and analyzed under constant air flow for potential MNM release. We demonstrated and quantified the presence of TiO_2_ on the outer layer of each face mask with Ti mass fractions ranging from 80 and 4870 µg/g of mask material, which is within the range reported in previous studies. TiO_2_ particles detected on all face masks exhibited a polydisperse size distribution on the mask surface, with smaller particle sizes observed in the fraction leached into the supernatant after 48 h of agitation in liquid ethanol. Only a small fraction of Ti (< 1% of the available Ti present) was released from Mask 1 and Mask 2, whereas approximately 53% of the total Ti was released from Mask 3. This corresponded to Ti levels ranging from < 1 and 37 µg/g for all three face masks.

Further investigation in-situ enables the evaluation of particle release in air from face masks under a worst-case scenario by testing a single particle-containing layer, under continuous air flow and vigorous agitation. The three masks selected were subsequently tested under the stated conditions. Results demonstrated that continuous air flow alone was not sufficient to dislodge particles, while particles below 100 nm were detected with inline agitation for Mask 1 and Mask 3 only, with 130 and 520 #/cm^3^ detected respectively. Further analysis of the shed particles did not indicate the presence of TiO_2_, but showed the presence of Ca, Cu and Si containing particles, with no high aspect ratio fibres detected. At a minimum, this study provides evidence that dislodging particles from the masks is difficult and that resuspension of either particles added to the mask or captured by the mask is unlikely. Nevertheless, additional reproducible data covering a broader range of face masks and MNMs is needed to further support these findings and better inform inhalation exposure risk assessments. The current findings also highlight the need for a standardized method to evaluate airborne particle shedding, enabling consistent comparisons across face masks and studies.

Definitive conclusions remain challenging, given that the low rates of particle shedding make airborne measurement difficult. This, combined with the potentially high toxicity of inhaled nanomaterials adds uncertainties to risk assessments. These findings further emphasize the importance of standardized nanomaterial integration processes in face masks manufacturing. Overall, the low TiO_2_ shedding potential observed in this work adds to the body of evidence relating to inhalation exposure to MNM indicating that shedding is unlikely.

## Supplementary Information

Supplementary Material 1

## Data Availability

The datasets generated during and/or analyzed during the current study are available from the corresponding author on reasonable request.
