# Supplementary material for: Quantification and characterization of manufactured nanomaterials shed from face masks
Source: Sci Rep. 2026 Feb 3;16:5416. doi: 10.1038/s41598-025-34482-6 (PMC12886907; doi:10.1038/s41598-025-34482-6)
Supplement: Supplementary file 1 — Supplementary Material 1 [file 41598_2025_34482_MOESM1_ESM.docx]

Quantification and characterization of manufactured nanomaterials shed from face masks

R. Mehri^1^, Z. Gajdosechova^1^, T.A. Sipkens^1^, G.J. Smallwood^1^, A.M. Belknap^2^, D. Vladisavljevic^2^, J.C. Corbin^1^

^1^ Metrology Research Centre National Research Council Canada, Ottawa, Canada

^2^ New Substances Assessment and Control Bureau, Health Canada, Ottawa, Canada

# Facemasks


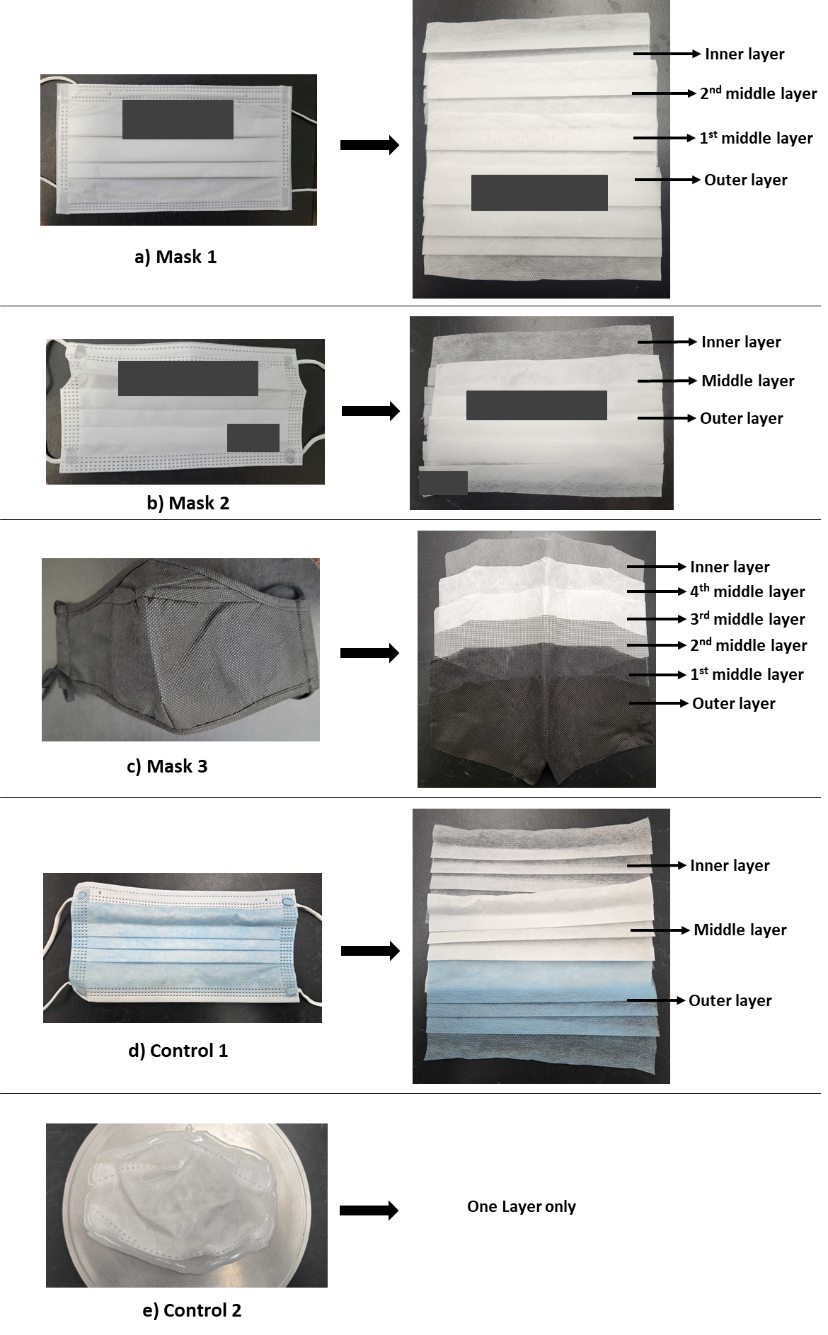


Fig S1: Description of the three facemasks and 2 controls selected. Mask 1 and 2 are disposable face masks made of 4 and 3 layers respectively, while Mask 3 is a reusable and washable face mask claimed to contain 5 layers. Control 1 is a surgical facemask made of 3 layers, while Control is composed of 1 layer only. For Mask 3, the composition of the outer layer was not disclosed by the manufacturer. Although Mask 3 is claimed to be composed on 5 layers, 6 layers were found when separating the facemask. Mask 1 and 2 claim to contain TiO_2_, while Mask 3 was claimed to have been coated with titania and silver on the outer layer. No claim was associated with Control 1 and Control 2.


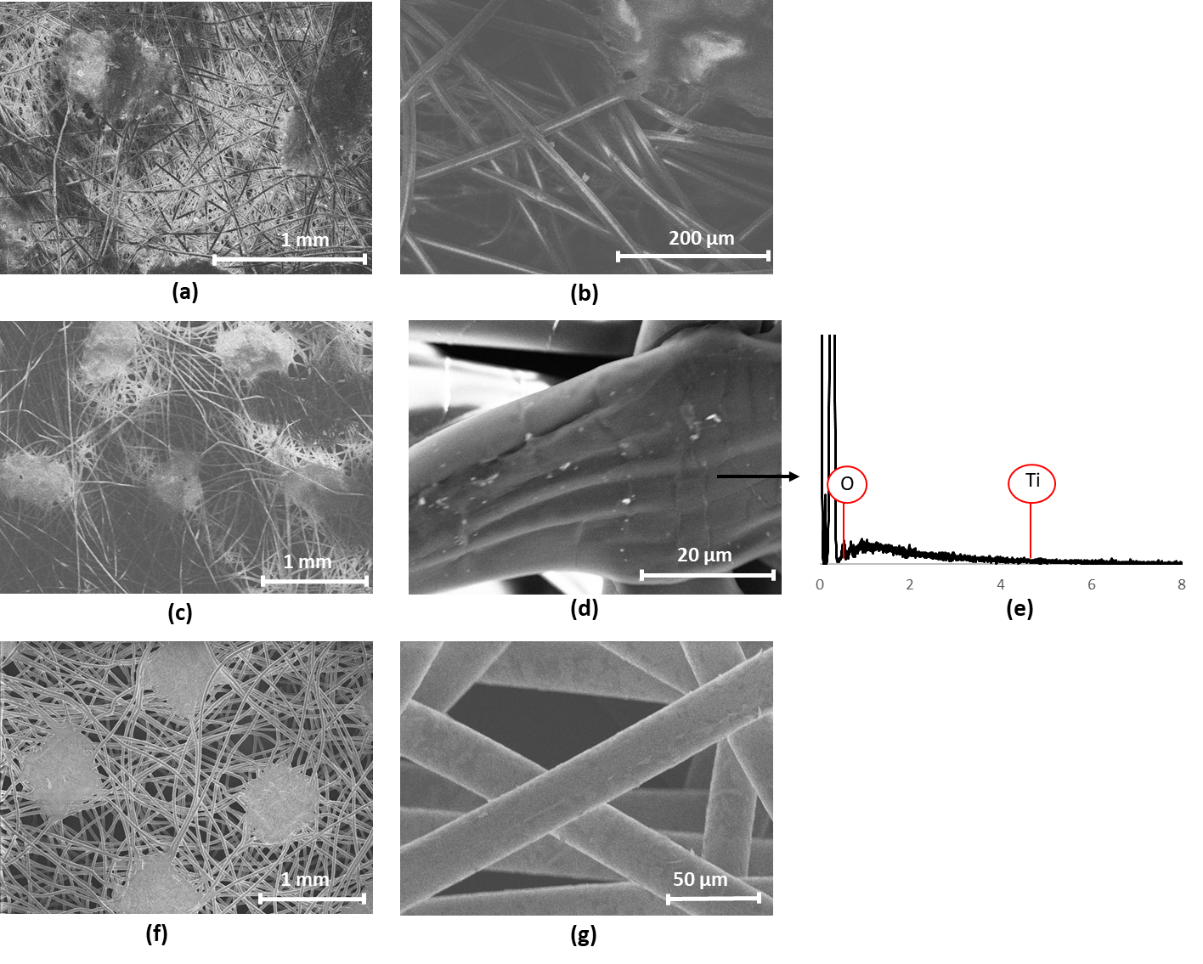


Fig S2: SEM images of the inner layer of (a) Mask 1, (c) Mask 2 and Mask 3 (f) with a higher magnification and EDX elemental map for Ti shown in (b), (d) and (g) respectively. The corresponding sum spectrum of the EDX elemental mapping is shown in (e) for Mask 2 only, where particles were observed on the inner layer.

# ICP-MS settings and instrument optimization

## Dissolved metal analysis

The Agilent 8900 ICM-MS/MS (Agilent Technologies, Santa Clara, CA, USA) was equipped with a standard sample introduction system consisting of a MircoMist glass concentric nebulizer, a quartz spray chamber, and a quartz torch with 2.5 mm id injector. The interface was equipped with a nickel-plated copper sampling cone and nickel skimmer cone. The instrument was operated in MS/MS mode using a mixed cell gas containing oxygen (O_2_) and hydrogen (H_2_) for detection of Ti. The method was applied to resolve the isobaric interferences, mainly arising from ^48^Ca and the matrix-based polyatomic interferences, such as those derived from C, S, and P. Q1 was set to *m/z* 48 (the mass of the precursor ^48^Ti^+^ ion) and Q2 was set to *m/z* 64 (the mass of the target product ion ^48^Ti^16^O^+^). The introduction of O_2_ in the reaction/collision cell led to formation of ^48^Ti^16^O^+^ product ion, and added H_2_ facilitated formation of ^48^Ca^16^O^1^H^+^, thereby eliminating interference on ^48^Ti^16^O^+^ by ^48^Ca^16^O^+^. The instrument was optimized daily for maximum sensitivity and stability with cerium oxide ratios of < 1% and doubly charged ions (^70^Ce^+^ / ^140^Ce^++^) < 2%. An online internal standard of scandium (Sc) and rhodium was continuously mixed with calibration standards and samples during the analysis. Monitored Sc isotope (*m/z* 45 → 61) and Rh isotope (*m/z* 103) were used to compensate for possible instrument instability and matrix effects by calculating the ratio between the *m/z* of the element of interest and the internal standard. Quantitation was performed using the external calibration method; quality control samples of medium concentration, as well as blank, were measured every 13 samples.

## Leached particle analysis

In SP-ICP-MS mode, the instrument’s introduction system was identical to the one used for dissolved metal analysis with the exception of a quartz torch with 1.0 mm id injector. The analysis was performed in time-resolved analysis (fast TRA) mode, using a dwell time of 0.1 ms per point without settling time between measurements. The flow rate was measured at the start and end of each sequence and was typically between 0.30 to 0.35 mL/min. The instrument was operated in the same MS/MS mode as for total metal analysis and optimization was performed the same way. The raw data were exported and processed with the SPCal software (version 1.3.2) ^1^ using automatic threshold detection with 5-sigma criteria for Gaussian filtering and Formula C for Poisson filtering.

# Detailed results of the face mask characterization by ICP-MS

Table S1: Average Ti mass fractions (µg/g of mask) from ICP-MS and the associated uncertainties (k = 1) before (Original) and after 48 hours of agitation in ethanol (After agitation). Repeats, n, referred to the number of repeat measurements on a single mask, while replicates correspond to different masks. Combined rows show weighted averages over the replicates, while the portion released provides and estimates of the fraction of particles released after agitation in liquid.

|  |  | **Original** | | **After agitation** | | |
| --- | --- | --- | --- | --- | --- | --- |
| **Mask** | **Replicate** | **Repeats, *n*** | **Ti Mass fraction ± SD [µg/g]** | **Repeats, *n*** | **Ti Mass fraction ± SD [µg/g]** | **Portion released** |
| **Mask 1** | **Combined** | **-** | **4290 ± 550** | **-** | **-** |  |
|  | 1 | 5 | 4070 ± 770 | - | - |  |
|  | 2 | 5 | 4290 ± 860 | 3 | 4290 ± 690 | < 1% |
|  | 3 | 5 | 4870± 2105 | - | - |  |
| **Mask 2** | **Combined** | **-** | **1940 ± 40** | **-** | **-** |  |
|  | 1 | 3 | 2200 ± 73 | - | - |  |
|  | 2 | 3 | 1660 ± 45 | 3 | 1680 ± 65 | < 1% |
|  | 3 | 3 | 2230± 110 | - | - |  |
| **Mask 3** | **Combined** | **-** | **95 ± 5** | **-** | **-** |  |
|  | 1 | 4 | 160 ± 28 | - | - |  |
|  | 2 | 4 | 80 ± 6 | 3 | 43 ± 7 | ~ 53% |
|  | 3 | 4 | 95 ± 34 | - | - |  |
| **Control 1** | **Combined** | **-** | **192 ± 25** | **-** | **-** |  |
|  | 1 | 3 | 183 ± 34 | - | - |  |
|  | 2 | 3 | 217 ± 62 | 3 | **LOD** | - |
|  | 3 | 3 | 186 ± 44 | - | - |  |
| **Control 2** | **1** | 3 | **LOD** | **-** | **-** | **-** |

LOD: below limit of detection < 0.464 µg/g, SD: standard deviation

*n*: Number of subsamples for each replicate.

# Validation of shedding experimental setup

In order to validate the experimental setup proposed to assess particle shedding, Control 2 facemask was used and separately loaded with sodium Chloride particles using an automated filter tester (8310, TSI Inc., USA) following the guidelines of the National Institute for Occupational Safety and Health (NIOSH) standard testing procedure ^2^. This test procedure is used to determine the particle filtration efficiency of non-powered, air-purifying respirators. Loading was performed at 85 L/min for a duration of 56 minutes with a mass concentration of ~20 mg/m^3^ resulting in a loading of approximately 80 mg. Although particle size distribution was not verified directly, green line media was used to ensure the filter tester would provide particles within the required range as outlined in the NIOSH standard procedure. Therefore, the particles loaded were assumed to have a geometric mean diameter (GMD) and geometric standard deviation (GSD) of 75 ± 20 nm and 1.8 respectively. Since the automated filter tester is equipped with photometers, calibrated to provide particle mass, we used the Hatch–Choate analysis ^3,4^ to calculate the particle number concentration *N_conc_* as follows:

| $\text{N}_{\text{conc}}\text{ =}\frac{\text{M}_{\text{conc}}}{\frac{\text{πρ}_{\text{eff}}}{\text{6}}\left[ \text{GMD }\text{e}^{\text{1.5}\text{(ln} \text{GS}\text{D)}^{\text{2}}} \right]^{\text{3}}}$ | (S1) |  |
| --- | --- | --- |

where *M_conc_* is the particle mass concentration expressed in mg/m^3^, and $\text{ρ}_{\text{eff}}$ represents the effective density of the NaCl particles. Assuming spherical particles, effective density can be taken as the material density of 2160 kg/m^3^. Based on the following analysis, the total particle number *N* deposited onto the filter is estimated based on:

| $\text{N}_{\text{loaded}}\text{=}\text{N}_{\text{conc}}\text{Qt}$ | (S2) |  |
| --- | --- | --- |

where *Q* represents the loading flow rate and *t* the loading time.

A pristine unloaded Control 2 (here referred to as the negative (-) control) and the loaded Control 2 (here referred to as the (+) control) facemasks were tested consecutively in the experimental setup shown in Figure 2 with concurrent inline agitation. In both cases, the inside of the facemask (in contact with the skin) was facing the incoming airflow, opposite to the loading configuration.

Released particles detected by the downstream CPC are shown in Figure S3. As expected, no particles were detected with the negative control. When using the positive control, a burst of particles released from the facemask was detected within the first 5 minutes of the test run. Minimal particle concentration (10-70 #/cm^3^) was observed for the remainder of the test run, resulting in an average particle concentration of approximately 200 #/cm^3^.


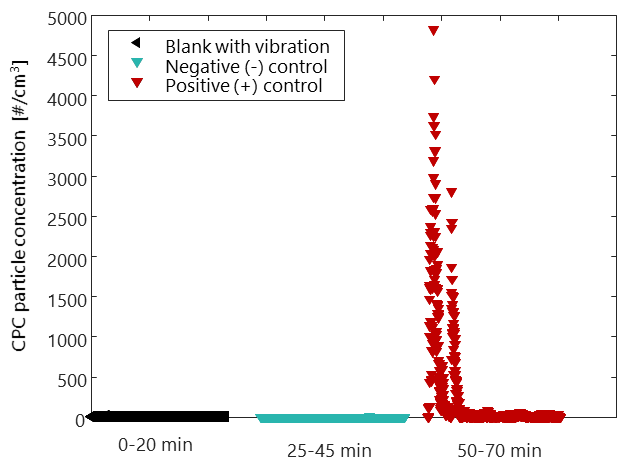


Fig S3: Number particle concentration measured using the downstream CPC for a blank plate, an unloaded Control 2 facemask (negative control) and an NaCl loaded Control 2 facemask (positive control). All tests were performed with inline agitation for a 20-minute period.

Similarly, applying, Eq. (S2), the total number of particles released from the loaded facemask can be calculated and used to determine the percent of resuspension as follows:

| $\text{Resuspension }\left( \text{\%} \right)\text{=}\frac{\text{N}_{\text{loaded}}}{\text{N}_{\text{released}}}$ | (S3) |  |
| --- | --- | --- |

In this validation study, although a burst of particles was released from Control 2, resuspension was found to be negligeable with a value of 0.00006% assuming a loaded particle size distribution with a GMD and GSD of 75 nm and 1.8 respectively.

# References

1 Lockwood, T. E., Gonzalez de Vega, R. & Clases, D. An interactive Python-based data processing platform for single particle and single cell ICP-MS. *Journal of Analytical Atomic Spectrometry* **36**, 2536-2544 (2021). <https://doi.org:10.1039/D1JA00297J>

2 NIOSH. (2019). Determination of Particulate Filter Efficiency Level for N95 Series Filters Against Solid Particulates for Non-Powered, Air-Purifying Respirators, TEB-APR-STP-0059.

3 Hinds, W. C. & Zhu, Y. *Aerosol technology: properties, behavior, and measurement of airborne particles*. (John Wiley & Sons, 2022).

4 Corbin, J. C. *et al.* Systematic experimental comparison of particle filtration efficiency test methods for commercial respirators and face masks. *Scientific Reports* **11**, 21979 (2021). <https://doi.org:10.1038/s41598-021-01265-8>
